# Epidemiological analysis of biofilm-forming methicillin-resistant *Staphylococcus aureus* clinical isolates

**DOI:** 10.3389/fpubh.2026.1783787

**Published:** 2026-05-05

**Authors:** Fethi Ben Abdallah, Rihab Lagha, Fehmi Boufahja, Wejdene Mansour

**Affiliations:** 1 Unité de Recherche: Génomique, Biotechnologie et stratégies antivirales, Institut Supérieur de Biotechnologie, University of Monastir, Monastir, Tunisia; 2 Laboratoire de Biophysique Métabolique et Pharmacologie Appliquée, Faculté de Médecine Sousse, University of Sousse, Sousse, Tunisia; 3 Biology Department, College of Science, Imam Mohammad Ibn Saud Islamic University (IMSIU), Riyadh, Saudi Arabia

**Keywords:** (GTG)_5_-PCR, biofilm, BOXA1R-PCR, crystal violet, genetic variability, methicillin-resistant *Staphylococcus aureus*, SCC*mec* type

## Abstract

**Introduction:**

Methicillin-resistant *Staphylococcus aureus* (MRSA) remains a significant global concern in healthcare and community environments, posing serious risks to patients due to its ability to form biofilm. Monitoring and spread control of epidemic MRSA clones require robust epidemiological typing methods.

**Methods:**

In this study, 30 MRSA isolates associated with significant morbidity were recovered from King Abdulaziz Specialist Hospital, Taif, Saudi Arabia. The strains were identified using the Vitek 2 automated system. The ability of MRSA to form biofilm on a polystyrene surface was evaluated by the crystal violet method. Genetic diversity of the strains was assessed using three methods: repetitive PCR based on (GTG)_5_, BOXA1R sequences, and multiplex PCR of the staphylococcal cassette chromosome *mec* (SCC*mec*).

**Results:**

Out of the 30 MRSA isolates, 29 strains were both highly positive (40%) and low-grade positive (56.66%) biofilm producers. Molecular epidemiology based on multiplex PCR of SCC*mec* showed that 10% of the isolates harbor each of SCC*mec* IVa and V. While 13.33% of the strains harbor the SCC*mec* II. In addition, 20% of the isolates were commonly associated with community-acquired, in contrast to 13.33% that were commonly associated with hospital-acquired infections. However, the remaining 66.66% of isolates were not classified into the tested SCCmec types. PCR genomic fingerprinting revealed high genetic variability of MRSA. (GTG)_5_ and BOXA1R-PCR generated 26 and 28 clusters with a discriminatory index of 0.99 at 90% similarity.

**Conclusion:**

MRSA isolates exhibited a high ability to produce biofilm, which can pose a serious public health problem. The quantification of biofilm in different clonal lineages is of great importance to develop effective antimicrobial policy and enhance biofilm management during infection. MRSA strains demonstrated significant genetic variability, indicating substantial genetic diversity. (GTG)_5_ and BOXA1R-PCR molecular typing methods are reliable for the epidemiological tracking of highly biofilm-forming MRSA strains in hospital environments and can provide essential insights into controlling the spread of MRSA infections.

## 1 Introduction

Methicillin-resistant *Staphylococcus aureus* (MRSA) represents a significant pathogen responsible for infections acquired both in the community and in healthcare settings. While it commonly leads to mild conditions, particularly involving the skin or soft tissues (1). This bacterium is also capable of causing severe diseases such as pneumonia, osteomyelitis, brain abscesses and sepsis. These serious infections are associated with morbidity, financial costs, and possible mortality (2). Given that MRSA constitutes one of the primary sources of enduring human infections and its pathogenic impact continues to pose substantial challenges worldwide.

Biofilms are structured microbial communities in which cells adhere to surfaces or to each other (3). In MRSA, especially hospital-acquired strains, biofilm formation represents a major virulence determinant. These surface-associated communities play a critical role in the development of catheter-related bloodstream infections, the colonization of medical devices, extensive tissue injury, and the persistence of nosocomial infections (4). Bacteria in biofilms can withstand antibiotic concentrations 10 to 10,000 times higher than those required to eliminate their planktonic forms (5). Biofilm development in *S. aureus* generally proceeds in two major stages. The initial step involves bacterial attachment to the surface, using capsular polysaccharide/adhesion. Then, the formation of a multilayered biofilm, through bacterial multiplication and the production of the polysaccharide intercellular adhesin, represents the second step. The synthesis of the capsular polysaccharide/adhesion and the polysaccharide intracellular adhesion was regulated by the intracellular adhesion (ica) locus (6). In addition, the development of biofilm by *S. aureus* was controlled by the accessory gene regulator (agr) quorum-sensing system (6).

The emergence of MRSA remains a major global concern in healthcare and community environments. It has been reported that children, as carriers, contribute to the dissemination of *S. aureus* across community and hospital settings. Monitoring and spread control of epidemic MRSA clones require robust epidemiological typing methods. Different molecular approaches, such as SCC*mec* typing and multi-locus sequence typing (MLST) (7), were used to determine the genetic relatedness between isolates of clinical relevance. Rep-PCR genomic fingerprinting methods found that (GTG)_5_-PCR enabled rapid molecular typing of *E. coli* (8). In addition, (GTG)_5_-PCR and BOXA1R-PCR have been considered suitable for genotyping *K. pneumoniae* (9).

This work aimed to investigate the biofilm-forming ability of MRSA clinical isolates. Furthermore, the MRSA epidemiology was analyzed using SCC*mec* typing and Rep-PCR genomic fingerprinting based on (GTG)_5_- and BOXA1R molecular markers.

## 2 Materials and methods

### 2.1 Bacterial strains

Thirty clinical isolates of MRSA associated with significant morbidity were randomly collected from King Abdulaziz Specialist Hospital, Taif, Saudi Arabia. Isolates of *S. aureus* were identified as outlined previously (10). The Vitek 2 automated system (bioMérieux, Durham, NC, USA) was used to identify the phenotype of methicillin resistance as described by the British Society for Antimicrobial Chemotherapy (BSAC, V12, 2013). Isolates that showed minimum inhibitory concentration (MIC) > 2 mg/L for oxacillin and >4 mg/L for cefoxitin were regarded as methicillin resistant.

MRSA strains were recovered from several infection sites, such as surgical site infection (SSI, *n* = 4), skin and soft tissue (SST, *n* = 12), blood (*n* = 1), nasal (*n* = 8), and burn (*n* = 5).

### 2.2 Biofilm formation

MRSA isolates biofilm formation was established using the 96-well microtiter plate method, previously detailed (11). Briefly, overnight cultures of bacteria were cultivated in Tryptic Soy Broth (TSB; Pronadisa, Spain) at 37 °C, and the optical density at 600 nm was measured (OD₆₀₀ = 1). Overnight cultures were diluted 1:100 in TSB with 2% (w/v) glucose, and 200 μL of the resulting suspension was added to U-bottomed 96-well microtiter plates (Nunc, Roskilde, Denmark). Three replicate experiments of each strain were conducted, and wells of sterile TSB served as negative controls. Planktonic cells were discarded following incubation at 37 °C for 24 h, and wells were washed twice using PBS (7 mM Na₂HPO₄, 3 mM NaH₂PO₄, and 130 mM NaCl, pH 7.4) to eliminate non-adherent bacteria, and the plates were air-dried. The adherent biofilm cells were fixed in 95% ethanol, stained using 1% crystal violet (Merck, France) for 5 min, and washed three times with 300 μL of distilled water. After drying, the optical density at 570 nm (OD₅₇₀) was measured using an automated Multiskan microplate reader (GIO. DE VITA E C, Rome, Italy). *S. aureus* ATCC 25923 was used as a positive control. Biofilm production was defined as highly positive (OD₅₇₀ ≥ 1), low-grade positive (0.1 ≤ OD₅₇₀ < 1), or non-biofilm producer (OD₅₇₀ < 0.1) (11).

### 2.3 Molecular analysis of MRSA isolates

#### 2.3.1 Multiplex PCR of the staphylococcal cassette chromosome

The major types and subtypes of staphylococcal cassette chromosome mec (SCC*mec*) were identified, in triplicate, by multiplex PCR assay (12). PCR mixture was performed in a total volume of 50 μL, including 25 μL of master mix (Promega, Madison, WI, USA), 5 μL of primer mix (2 mM for each primer) (Table 1), and 50 ng of genomic DNA. The reaction was carried out according to the following program: initial denaturation at 95 °C for 15 min, followed by 30 cycles of denaturation at 94 °C for 30 s, 57 °C for 1.5 min, and 72 °C for 1.5 min, ending with a final extension at 72 °C for 10 min. *S. aureus* ATCC 25923 was used as a positive control. PCR amplicons were analyzed on 1% agarose gel stained with ethidium bromide (0.5 mg/mL), photographed using Bio-Rad Gel Doc 2000 (Germany), and their sizes were determined with a 100 bp molecular size marker (Bio-Rad). The SCC*mec* type was determined based on the band pattern obtained.

**Table 1:** Primers used for SCC*mec*, (GTG)_5_, and BOXA1R typing of MRSA isolates.

Primer	Sequence	Gene	Size (bp)
Type I	For: GCTTTAAAGAGTGTCGTTACAGG Rev.: GTTCTCTCATAGTATGACGTCC	ORF E008 of strain NCTC10442	613
Type II	For: GATTACTTCAGAACCAGGTCAT Rev.: TAAACTGTGTCACACGATCCAT	*kdpE* of strain N315	287
Type III	For: CATTTGTGAAACACAGTACG Rev.: GTTATTGAGACTCCTAAAGC	J1 region of SCC*mec* type III	243
Type IVa	For: GCCTTATTCGAAGAAACCG Rev.: CTACTCTTCTGAAAAGCGTCG	ORF CQ002 of strain CA05	776
Type IVb	For: AGTACATTTTATCTTTGCGTA Rev.: AGTCATCTTCAATATGGAGAAAGTA	J1 region of SCC*mec* type IVb	1,000
Type IVc	For: TCTATTCAATCGTTCTCGTATT Rev.: TCGTTGTCATTTAATTCTGAACT	IVc element of strain 81/108	677
Type IVd	For: AATTCACCCGTACCTGAGAA Rev.: AGAATGTGGTTATAAGATAGCTA	CD002 in type IVd	1,242
Type IVh	For: TTCCTCGTTTTTTCTGAACG Rev.: CAAACACTGATATTGTGTCG	J1 region of strain HAR22	663
Type V	For: GAACATTGTTACTTAAATGAGCG Rev.: TGAAAGTTGTACCCTTGACACC	ORF V011 of strain JCSC3624	325
mecA	For: TCCAGATTACAACTTCACCAGG Rev.: CCACTTCATATCTTGTAACG	*mecA* gene	162
Sa442	For: AATCTTTGTCGGTACACGATATTCTTCACG Rev.: CGTAATGAGATTTCAGTAGATAATACAACA	Species-specific target	108
(GTG)_5_	GTGGTGGTGGTGGTG	(GTG)5	
BOXA1R	CTACGGCAAGGCGACGCTGACG	BOXA1R	

#### 2.3.2 Genotyping of *S. aureus* isolates using (GTG)_5_ and BOXA1R methods

Genotypic patterns of MRSA isolates were identified by repetitive sequence-based PCR using the (GTG)₅ and BOXA1R primers (13). All the reactions were prepared in a volume of 25 μL that included 12.5 μL GoTaq® Green Master Mix (Promega, Madison, WI, USA), 1 μL (10 pmol) of either the (GTG)₅ or BOXA1R primer (Table 1), and 50 ng of genomic DNA. The PCR reaction began with the initial denaturation at 95 °C for 5 min, followed by 40 successive cycles of denaturation at 94 °C for 1 min, annealing at 52 °C for 1 min, and extension at 72 °C for 2.5 min. The final extension was done at 72 °C for 7 min. *S. aureus* ATCC 25923 was used as a positive control. Agarose gel electrophoresis of amplified DNA fragments was carried out on 1.5% ethidium bromide-stained agarose gels (0.5 mg/mL) and visualized with a Bio-Rad Gel Doc 2000 (Germany). An estimated size of the bands was obtained by applying a 100 bp DNA ladder (GeneDireX, Germany). Experiments were carried out three times to ensure the reproducibility of the result.

#### 2.3.3 Cluster analysis

The banding profiles produced by BOXA1R and (GTG)_5_-PCR were analyzed using PyElph version 1.4. The dendrograms were constructed from the BOXA1R-PCR and (GTG)₅-PCR fingerprints using the Dice coefficient and the UPGMA clustering method (tolerance 1%).

#### 2.3.4 Discriminatory index

Simpson’s index of diversity (discriminatory index (D)) was determined using the following formula (14):

$$D=1-\frac{1}{N-1}\sum_{j=1}^{s}n_{j}(n_{j}-1)$$

*N* is the total number of isolates in the sample population, *s* is the total number of types described, and *n_j_* isthe number of strains belonging to the *j*th type. Simpson’s index of diversity ranges from 0 to 1. A value of 1 is highly discriminatory, and a value of 0 is not discriminatory.

### 2.4 Statistical analysis

Statistical analysis was conducted using analysis of variance (ANOVA). The Spearman’s correlation (*rₛ*) and their significance (*p*) were conducted using IBM SPSS Statistics for Windows, Version 20 (Released 2011; IBM Corp., Armonk, New York, United States).

## 3 Results

### 3.1 Biofilm formation

**Figure 1:**
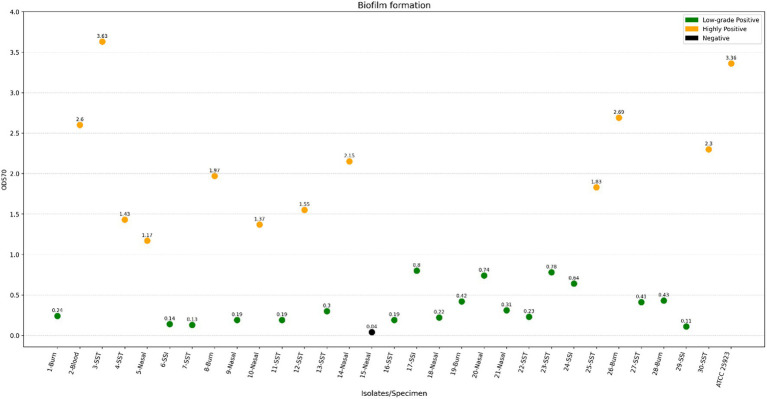
Biofilm formation by methicillin-resistant *Staphylococcus aureus*.

Thirty MRSA isolates were screened for biofilm production. Among them, 29 strains were able to produce biofilm on polystyrene surfaces with OD570 values ranging from 0.11 to 3.63. According to Figure 1, 11 isolates were highly positive producers. However, 18 isolates were low-grade positive producers with an OD570 value going up to 0.87. The strain number 15 provided from the nasal specimen was biofilm negative. *S. aureus* ATCC 25923, which was used as a control, was highly positive for biofilm formation. Statistical analysis indicates that specimen type does not significantly influence the formation of biofilm (*F* = 0.677; *p* = 0. 273).

### 3.2 SCC*mec* typing

**Figure 2:**
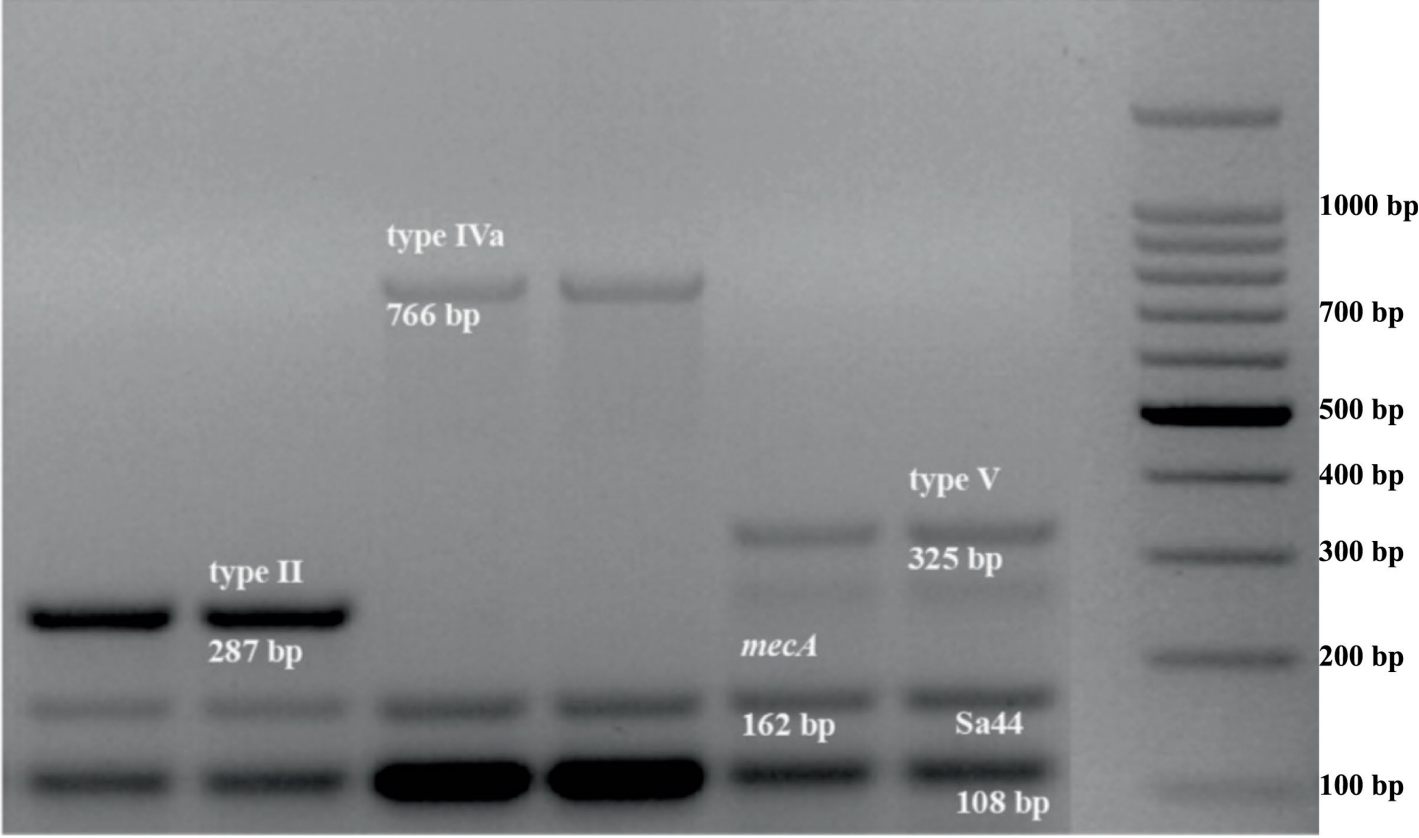
Multiplex PCR analysis of SCC*mec* in MRSA isolates.

Multiplex PCR presented in Figure 2, showed that all the strains were positive for Sa442, and the *mec*A genes confirmed that the isolates are MRSA. SCC*mec* typing revealed that strains 5, 10, and 20 (10% of the isolates) harbor the SCC*mec* type IVa. However, strains 2, 3, and 23 (10% of the isolates) harbor SCC*mec* type V, indicating that these six strains are commonly associated with community-acquired MRSA (CA-MRSA). Additionally, 13.33% of the isolates (8, 14–16, and) are commonly associated with hospital-acquired MRSA (HA-MRSA), carrying SCC*mec* type II. The analysis also showed that 20 isolates (66.66%) are non-typable and therefore are not classified into the tested SCC*mec* types. Statistical analysis revealed no significant correlation between SCC*mec* typing and biofilm formation (*r_s_* = 0.321; *p* = 0.197).

### 3.3 BOXA1R and (GTG)_5_ methods for MRSA genotyping

**Figure 3:**
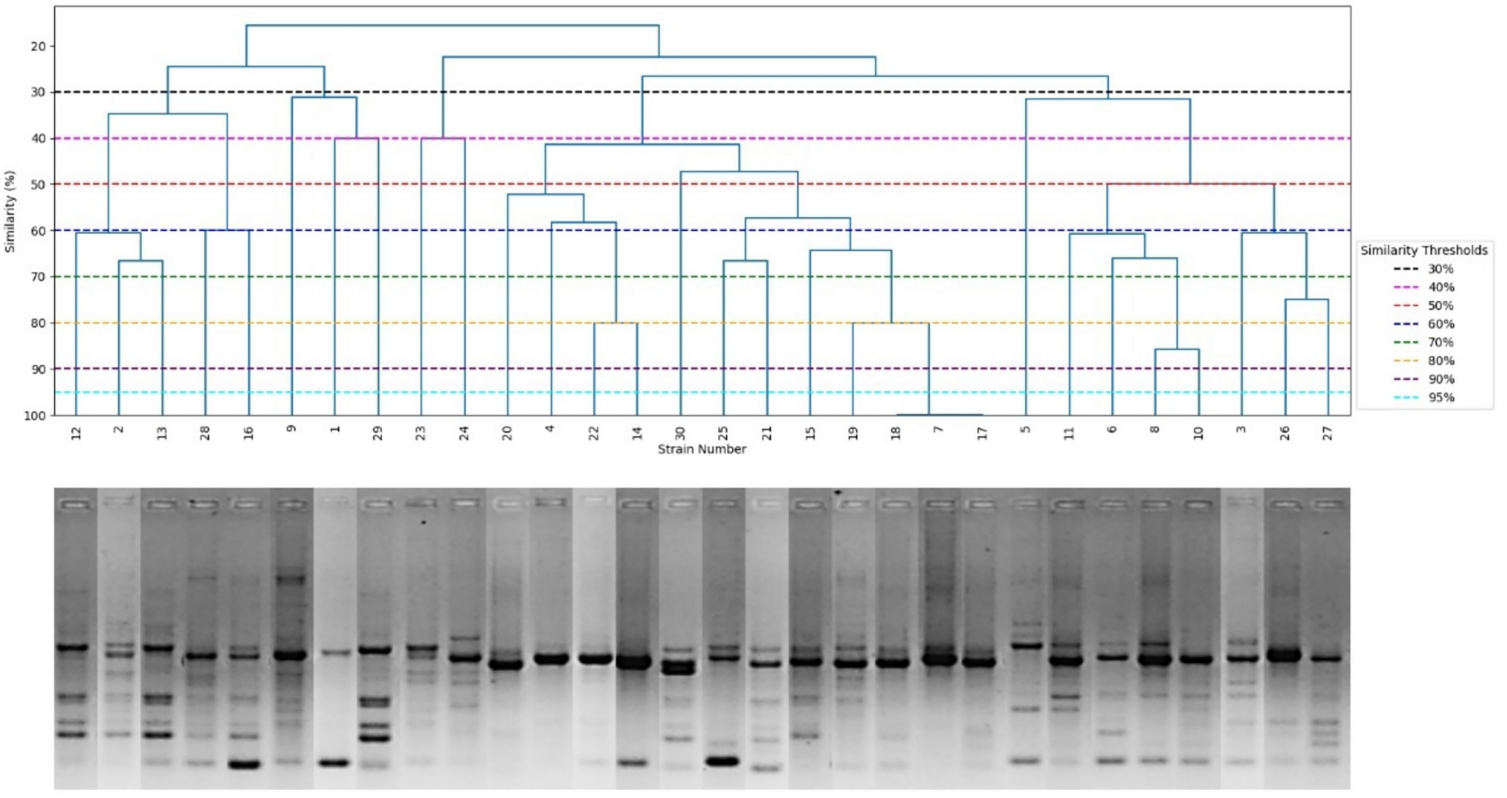
UPGMA dendrogram generated using the Dice coefficient from (GTG)_5_-PCR fingerprints of MRSA isolates.

**Figure 4:**
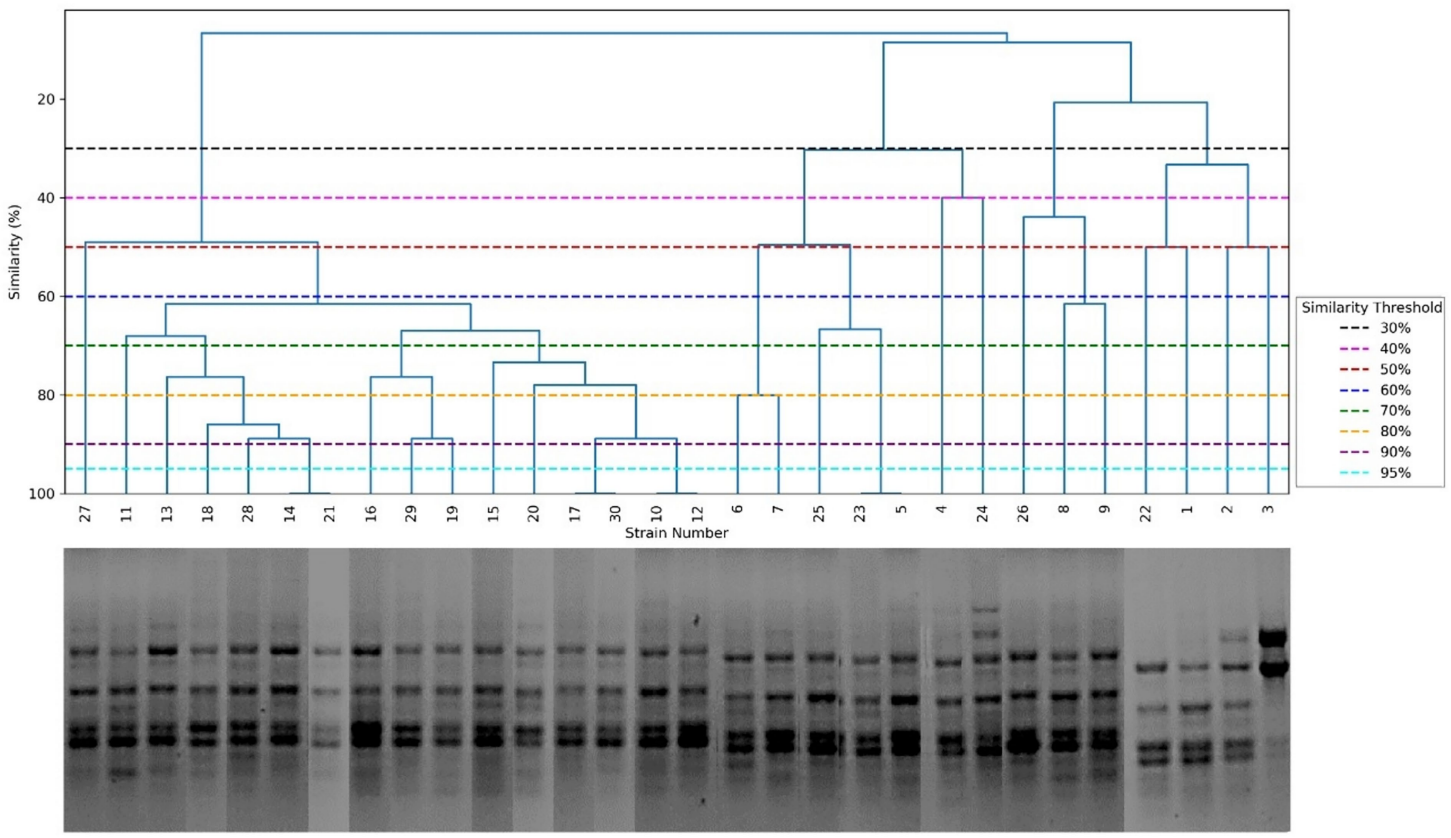
UPGMA dendrogram generated using the Dice coefficient from BOXA1R-PCR fingerprints of MRSA isolates.

Molecular typing of MRSA isolates using the (GTG)_5_-PCR and BOXA1R-PCR generated 2 to 15 and 4 to 11 bands, respectively, ranging from 150 to 1,500 bp (Figures 3, 4). Clusters were defined using arbitrary similarity thresholds of 50, 70, and 90%. As summarized in Table 2, the (GTG)_5_-PCR grouped the strains into 13, 24, and 28 clusters, with discriminatory indices of 0.90, 0.94, and 0.99, respectively. In contrast, the BOXA1R-PCR generated 10, 17, and 26 clusters, with corresponding discriminatory indices of 0.74, 0.93, and 0.99. Spearman’s rank correlation analysis demonstrated that no statistically significant correlation existed between the biofilm-forming capacity of the isolates and their genotyping profiles (*r_s_* = 0.078, *p* = 0.566 for (GTG)_5_; *r_s_* = 0.175; *p* = 0.348 for BOXA1R).

**Table 2:** Discriminatory indices of (GTG)_5_-PCR and BOXA1R-PCR in genotyping of MRSA isolates.

Genotyping method	Similarity %	Number of clusters	Cluster sizes	Discriminatory index
(GTG)_5_-PCR	50	13	4, 1, 3, 3, 1, 1, 1, 7, 4, 1, 2, 1, 1	0.90
	70	24	2, 1, 1, 1, 1, 1, 1, 1, 1, 1, 4, 2, 2, 1, 1, 1, 1, 1, 1, 1, 1, 1, 1, 1	0.94
	90	28	1, 1, 1, 1, 1, 1, 1, 1, 1, 1, 3, 1, 1, 1, 1, 1, 1, 1, 1, 1, 1, 1, 1, 1, 1, 1, 1, 1	0.99
BOXA1R-PCR	50	10	2, 2, 1, 3, 1, 2, 2, 1, 1, 15	0.74
	70	17	1, 1, 1, 1, 1, 2, 1, 1, 2, 1, 1, 1, 1, 6, 1, 5, 3	0.93
	90	26	1, 1, 1, 1, 1, 2, 1, 1, 1, 1, 1, 1, 1, 1, 2, 1, 1, 2, 1, 1, 1, 2, 1, 1, 1, 1	0.99

## 4 Discussion

MRSA infections represent a major public health concern worldwide, as this pathogen continues to be a leading cause of both hospital-acquired and community-acquired infections (1). MRSA, as one of the most common nosocomial pathogens, can cause a broad spectrum of infections, ranging from mild skin infections to severe abscesses, sepsis, endocarditis, osteomyelitis, and urinary tract infections (2). In this finding, the distribution of MRSA isolates varied across specimen types. Most of the strains (40%) were recovered from skin and soft-tissue samples, followed by nasal, burn, surgical-site infection, and blood samples, which confirm the high heterogeneity of this bacterium. This result is in line with the finding of Akanbi et al. (17) that revealed a high prevalence of MRSA in blood, wound, and urine samples. However, other strains were frequently isolated from surgical wound infections, eye swabs, and skin and soft tissue samples. In addition, Ahmadi et al. (18) highlighted the prevalence of MRSA in wound infections.

Biofilm formation is a virulence factor that contributes to MRSA infections and has become a special concern due to increasing resistance to antibiotics, which often leads to treatment failures and persistent infections (5). This work investigated the capacity of MRSA to develop biofilm through quantitative analyses using the crystal violet method. Results showed variation in biofilm-forming abilities, with 56.66% of the strains being low-grade positive, while 40% were highly positive. Previous reports indicated variation from 43 to 88% in the prevalence of biofilm formation among *S. aureus* (19). In this study, *in vitro* evaluation of biofilm formation in MRSA strains showed a prevalence of 96.66%, which is higher than the previously reported rate in Egypt (83.3%) (15) and Iran (38.7%) (16). In general, biofilm is accountable for more than 65% of nosocomial infections. In addition, about 80% of microbial infections promote the chronicity of *S. aureus* infections. Indeed, various staphylococcal diseases are related to the biofilm, such as skin and soft tissue infections, nasal colonization, endocarditis, and urinary tract infections (4). Moreover, biofilm formation represents a significant threat in urology, since it’s responsible for the long persistence of bacteria within the genitourinary tract (20). According to our study, the strong biofilm-forming ability observed in the investigated isolates further supports that MRSA is a predominant species in biofilm-associated infections.

In this study, statistical analysis showed no significant correlation between the origin of the strain and the biofilm formation. Despite the strains recovered from skin and soft tissue samples producing more biofilm than those isolated from nasal and burn specimens. Further, the only blood isolate formed a strong biofilm. However, strains originating from the surgical site infection produced only moderate biofilm. According to the finding of Silva et al. (21), MRSA from bacteremia and diabetic foot developed strong biofilm compared to those from osteomyelitis. It has also been shown that isolates from blood produce more biofilm than those from skin lesions, urinary tract infections, and sputum (22). However, another report revealed some MRSA blood isolates with low-level biofilm formation (23). This variability may reflect the influence of other factors, such as the virulence genes and the clonal lineages, on the biofilm formation ability of MRSA strains. The high potential of strains isolated from skin tissue to develop biofilm can be attributed to the fact that the biofilm’s mode of growth may enhance protection against antimicrobial agents routinely used in skin wounds (23). It is also important to emphasize that the presence of highly positive biofilm-producing MRSA strains in blood is particularly concerning, as these strains have the potential to disseminate to other sites and cause secondary infections, including infective endocarditis, septic arthritis, and osteomyelitis (24). This finding provided valuable data about the capacity of clinical MRSA isolates to form biofilm, which can be supported by larger studies that include a higher number of isolates.

A comprehensive understanding of circulating MRSA strains serves as a fundamental requirement for effective control and surveillance strategies. The dissemination of MRSA poses a significant threat to healthcare professionals and infection control personnel (25). Considering the substantial heterogeneity among Staphylococcal strains, the contribution of hospital environments in the development of nosocomial outbreaks warrants attention. Given the critical importance of MRSA strain transmission, implementing molecular approaches for epidemiological investigations of these strains proves essential (7). In this study, the SCC*mec* typing method was used for genotyping the MRSA isolates from Taif in Saudi Arabia. Multiplex PCR showed that 10% of the isolates harbor each of the SCC*mec* type IVa and SCC*mec* type V. While 13.33% of the strains harbor the SCC*mec* type II. Despite the small sample size, the most frequently detected SCC*mec* was type II, which is in discordance with a previous study in Saudi Arabia (26). A retrospective study of clinical MRSA isolates from Saudi Arabia, collected between 2023 and 2024, revealed that SCC*mec* type V was the predominant genotype (87.1%), indicating a rising prevalence of community-associated MRSA in both hospital and community environments (27). Moreover, study conducted in Northern Saudi Arabia showed that SCC*mec* types IVd (39%), IVc (27%), and V (24%) were most frequent, reflecting the circulating genetic diversity of MRSA clones in the region (28). Various studies have reported the variation in SCC*mec* types between countries or different centers within a country or even within the same region over time (29). Type IV SCC*mec* was predominant in Japan between 1979 and 1985, but after 1900, type II SCC*mec* dominated Japanese hospitals (30). Most Asian countries reported the dominant SCC*mec* type II. There is a high prevalence of SCC*mec* types IV and V among device associated strains (29, 31). This suggests that the dominant SCC*mec* type is dependent on various factors, such as the host, geographical location, study period, and patient groups.

Our results indicated that 20% of the isolates were commonly associated with community-acquired, in contrast to 13.33% that are commonly associated with hospital-acquired infections, suggesting the presence of MRSA in community settings. In addition, 20 isolates (66.66%) were non-typable, underscoring the limitations and reduced reliability of the SCC*mec* typing method in providing solid evidence for distinguishing the independent origins of healthcare-associated and community-acquired MRSA. Moreover, the multiplex PCR assay may not be able to detect new variants or non typable SCC*mec* cassettes with existing primers. Further, extreme polymorphisms may also explain the absence of SCC*mec* elements in some strains.

Genotyping is a relevant tool for nosocomial infection studies. In this work, Rep-PCR based on (GTG)_5_ and BOXA1R-PCR demonstrated high genetic diversity of investigated MRSA isolates. Indeed, at a similarity of 90%, (GTG)_5_- PCR generated 28 clusters for the 30 MRSA isolates, with a discriminatory index of 0.99. However, BOXA1R-PCR generated 26 clusters, with a discriminatory index of 0.99. Simpson’s index of diversity suggests that these methods were suitable for bacterial discrimination. Assessment of the number of clusters indicated that (GTG)₅-PCR was marginally more effective for MRSA discrimination than BOXA1R-PCR, which is in agreement with previous reports (9). Nevertheless, (GTG)₅-PCR generated fewer bands than BOXA1R-PCR. Importantly, the low number of bands in fingerprints can be discriminative in small genomic variations, highlighting a potential advantage of BOXA1R-PCR for discriminating closely related isolates (9, 32). In addition, this result corroborates the data of Mutlu et al. (33), showing that BOXA1R-PCR, as a fast and good molecular method, can be useful in finding the source of the disease outbreak and routine identification of the microorganism. Both (GTG)₅ and BOXA1R did not show a significant correlation with biofilm formation, indicating that the genetic variability of the strain did not affect the biofilm formation process. Biofilm formation is a complex, multifactorial process regulated by multiple genetic determinants, which may not be directly associated with SCC*mec* elements or overall genomic fingerprints. Additionally, phenotypic variability in biofilm production can occur even among genetically related strains due to differences in gene expression and environmental adaptation.

This preliminary finding provides valuable insights into the molecular epidemiology of MRSA in Taif, Saudi Arabia, and highlights the prevalence of specific MRSA strains and their biofilm profiles. This result could be essential for monitoring and addressing the spread of MRSA in healthcare settings.

## 5 Conclusion

This study highlights the prevalence, biofilm formation, and genetic diversity of MRSA isolates from Saudi Arabia. The majority of the isolates exhibited both strong and intermediate biofilm production capabilities, which can pose a serious public health problem since biofilm is considered a major virulence factor. The quantification of biofilm in different clonal lineages may contribute to the development of effective antibiofilm components and optimize biofilm management during infection. This study also shows that (GTG)_5_ and BOXA1R-PCR, as molecular typing methods, can be used for the epidemiological tracking of MRSA strains in hospital environments and can provide essential insight into controlling the spread of MRSA infections. However, the SCC*mec* typing method has proven inadequate and unreliable for distinguishing the independent origins of healthcare-associated and community-acquired MRSA. This study included only 30 isolates, while the small sample size limits the robustness of epidemiological and policy-related implications, the findings represent preliminary data emphasizing further investigations with a larger cohort. However, to better understand the epidemiological dynamics and transmission pathways of MRSA, future research should involve a larger multicenter study including isolates from different hospitals and geographic areas to improve representativeness power. In addition, the integration of higher-resolution molecular typing approaches such as spa typing, multilocus sequence typing (MLST), and whole-genome sequencing would allow more precise strain discrimination, improved inter-laboratory comparability, and deeper insight into evolutionary relationships.

## Data Availability

The original contributions presented in the study are included in the article/supplementary material, further inquiries can be directed to the corresponding authors.
